# SOX17-silenced HPAECs upregulate NF-κB-induced CXCL10 and CXCL11: implications for lymphocyte chemotaxis in SOX17-PAH

**DOI:** 10.1038/s41598-025-16418-2

**Published:** 2025-10-01

**Authors:** Abdul S. Mahomed, Anne Burke-Gaffney, Shahin Moledina, Quezia K. Toe, Dongmin Shao, Gregory J. Quinlan, Christopher J. Rhodes, James E. Pease, Stephen John Wort

**Affiliations:** 1 Faculty of Medicine, National Heart and Lung Institute, Imperial College London, SW3 6LY London, UK; 2 Paediatric Cardiology and National Paediatric Pulmonary Hypertension Service, Great Ormond Street Hospital for Children NHS Foundation Trust, London, UK; 3 National Pulmonary Hypertension Service, Royal Brompton Hospital, London, UK

**Keywords:** SOX17, Chemokines, Chemotaxis, Leukocytes, Pulmonary hypertension, Chemotaxis, Inflammation

## Abstract

Pulmonary arterial hypertension (PAH) is a progressive pulmonary vasculopathy characterized by extensive pre-capillary arterial remodeling, instigating increased pulmonary vascular resistance and eventual right heart failure. Rare mutations in the *SOX17* gene and common variants in the enhancer region are thought to predispose to PAH. Central to the PAH pathobiology is lung immune cell recruitment, orchestrated by the overproduction of chemokines (e.g. CXCL10) via the induction of NF-κB. Emerging evidence from murine models of *SOX17* impairment suggests perivascular leukocyte accumulation in the lung, likely due to disordered inflammatory mediator expression. Therefore, in the current study we assessed the role of SOX17 deficiency in human pulmonary artery endothelial cells (HPAECs) on selected inflammatory mediator release. Following a semi-quantitative array of 100 cytokines and chemokines, we identified markedly elevated CXCL10 and CXCL11 mRNA and soluble protein release in *SOX17*-silenced HPAECs (versus control siRNA-treated cells), driven by excessive NF-κB p65 activity. Further, we show that plasma CXCL10 levels are raised in a small cohort (*n* = 3) of carriers of pathogenic *SOX17* rare variants versus healthy controls. Finally, *SOX17* knockdown HPAEC supernatants mediated the in vitro migration of transfectants expressing CXCR3. Therefore, enhanced lymphocyte migration may be a pathomechanism of PAH due to *SOX17* loss, driven by a CXCL10/CXCL11/CXCR3 axis.

**Supplementary Information:**

The online version contains supplementary material available at 10.1038/s41598-025-16418-2.

## Introduction

Pulmonary arterial hypertension (PAH) is a progressive vasculopathy centred on extensive small intrapulmonary arterial remodeling, leading to increased pulmonary vascular resistance and eventual afterload-induced right ventricular failure^1^. Recently, case-control genome wide association studies suggest rare pathogenic variants in the SRY-related HMG-box (SOX17) gene, increase PAH risk, in addition to common variants upstream of the gene promoter^2–4^.

Indeed, insights are emerging into the pathophysiology of functional SOX17 loss in PAH and studies suggest that initial endothelial dysfunction may be a precursor for remodeling. Endothelial-specific knockout of *SOX17* or enhancer region knockout, in mice exposed to hypoxia or SUGEN5416/hypoxia exaggerates the development of pulmonary hypertension, as demonstrated by increased pulmonary arteriolar muscularization and endothelial cell proliferation, along with right ventricular hypertrophy and raised right ventricular systolic pressure^3,5,6^. Interestingly, recent studies suggest that endothelial-SOX17 deficiency may instigate increased pulmonary vascular inflammatory responses. Mice with endothelial-specific *SOX17* deletion, show increased perivascular accumulation of CD45^+^ cells, compared with wild-type (WT)^6^ and also increased lung infiltration of CD11b^+^ cells in hypoxic adult mice with *SOX17* pulmonary endothelial cell deletion versus WT^5^. Further, in hypoxia and tumor necrosis factor-alpha (TNFα)-treated HPAECs, nuclear NF-κB (nuclear factor kappa B) p65 is reduced following treatment with exosomes purified from SOX17-overexpressed human pulmonary artery endothelial cells (HPAECs) versus controls, suggesting that SOX17 may regulate NF-κB activity^7^.

In PAH, it is widely regarded that aberrant inflammatory responses are key molecular drivers of progressive vascular remodeling, in part due to excess NF-kB activation^8^. In particular, dysregulated expression of several chemokines has been implicated in the disease and are thought to facilitate the recruitment of immune cells to sites of pulmonary vascular lesions, among other roles^9,10^. For instance, some members of the CXC chemokine family, are shown to have raised serum concentrations in PAH patients, for example C-X-C motif chemokine ligand (CXCL)10 ^11^. Serum CXCL10 is also increased in systemic sclerosis (SSc)-PAH where it correlates positively with pulmonary hemodynamics, serum brain natriuretic peptide (BNP) and negatively with 6-minute walk distance (6MWD)^12^. Other chemokine family members include CXCL11, of which along with CXCL10, are small proteins (~ 8-9kD) with functional pleiotropy described in both health and disease, including angiogenesis, tumour formation and inflammation^13–17^. Additionally, both chemokines have well established roles in leukocyte migration, whereby they are shown to chemoattract T cells and macrophages via the CXCL10 and CXCL11 receptor, C-X-C motif chemokine receptor 3 (CXCR3)^18–22^.

In the present study, we aimed to investigate whether *SOX17* loss in HPAECs would result in excessive release of cytokines and chemokines, by semi-quantitatively assaying approximately 100 inflammatory markers. As such, here we identify that the chemokines, CXCL10 and CXCL11 are markedly secreted in *SOX17*-silenced HPAECs and that abnormal NF-κB p65 activity underlies their regulation. In turn, we show that supernatants from *SOX17* knockdown HPAECs promote in vitro migration of CXCR3 transfectants which is sensitive to a small molecule antagonist of the receptor.

## Materials and methods

### Cell culture

HPAECs were acquired from either PromoCell GmbH (Germany) or ATCC^®^ (USA) and were maintained in endothelial growth medium 2 (EGM2) containing 2% fetal calf serum (FCS) on cultureware pre-coated with bovine plasma fibronectin (10 µg/mL; Sigma-Aldrich) at 37 °C, supplied with 5% CO_2_. Multiple donors were used for experiments. The murine pre-B lymphocyte cell line L1.2 was maintained in RPMI containing 1 mg/mL Geneticin (G418). L1.2 lymphocytes were stably transfected with pCDNA3 containing the CXCR3A cDNA HA-tagged at the N-terminus, performed by electroporation, as previously described (L1.2-CXCR3 transfectants)^23,24^. Cells were cultured in 10 mM sodium butyrate for 24 h prior to experimentation.

### siRNA transfection

Silencing of *SOX17* and other genes in HPAECs was induced using siRNA, as previously described^25^. Briefly, HPAECs were transfected with 50nM ON-TARGETplus SMARTpool siRNA (Horizon Discovery, UK) against *SOX17* (siSOX17), *RELA* (siRELA), a combination of siRELA and siSOX17 or a non-targeting control pool (siControl), complexed with Lipofectamine RNAiMAX Transfection Reagent (Thermo Fisher Scientific, UK), in Opti-MEM™ I Reduced Serum Medium (Gibco, UK) for 14 h. Media was replaced with EGM2 following incubation with transfection mixes.

### RT-qPCR

250 ng total RNA was transcribed to cDNA using M-MLV Reverse Transcriptase (Thermo Fisher Scientific, UK) using a G-Storm GS1 Thermal Cycler (Labtech International, UK), according to manufacturer recommendations. RT-qPCR was carried out using, iTaq™ Universal SYBR Green supermix (Bio-Rad, UK) on a Corbett Rotor-Gene 6000 real-time PCR thermocycler (Qiagen, Germany). Relative changes in target gene (*SOX17*,* IL6*,* IL8*,* IL1RL1*,* NAMPT*,* PTX3*,* ENG*,* CCL2*,* CXCR3*,* CXCL10*,* CXCL11*,* ICAM1*,*VCAM1 a*nd *RELA*) expression was quantified using the 2^−ΔΔCt^ method with *ACTB* and *GAPDH* used as endogenous normalization controls. Forward and reverse primer sequences can be found in the online data supplement (Supplementary Table 1).

### Western immunoblotting

To confirm SOX17 protein knockdown and to determine levels of NF-kB p65/phosphorylated-NF-kB p65 post-siRNA transfection in HPAECs, cells were lysed with radioimmunoprecipitation assay buffer (Sigma-Aldrich, UK; supplemented with a protease and phosphatase inhibitor cocktail [Abcam, UK]). 30 to 40 µg of total protein was electrophoresed using 4–15% Mini-PROTEAN^®^ TGX Stain-Free™ precast polyacrylamide gels (Bio-Rad, UK) and subsequently electroblotted onto a nitrocellulose membrane (GE Life Sciences, UK). Membranes were then probed for anti-SOX17 (1:500 Abcam, UK). anti-NF-κB p65 (1:1000; CST, UK) or anti-Phospho-NF-κB p65 (Ser536; 1:1000; CST, UK), as well as anti-α-Tubulin (1:1000; CST, UK), to confirm equal protein loading. Protein bands were then detected using enhanced chemiluminescence (Odyssey Fc Imaging System [LI-COR Biosciences]).

### Cytokine and chemokine antibody array

Differential expression of soluble inflammatory mediator release from siControl and siSOX17-transfected HPAECs (24 h post-transfection) were determined using the Human XL Cytokine Array Kit (R&D Systems, ARY022B). Assays were performed according to the manufacturer’s instructions. Enhanced chemiluminescence detection was carried out using the Odyssey Fc Imaging System.

### Enzyme-linked immunosorbent assay (ELISA)

Quantification of soluble CXCL10 and CXCL11 expression in siControl, siSOX17, siRELA and siSOX17/siRELA supernatants (24 h post-transfection), was determined using Human DuoSet ELISAs (R&D Systems, UK), in accordance with instructions detailed by the manufacturer.

### Chemotaxis assay

Chemotaxis of L1.2-CXCR3 transfectants was assessed using a ChemoTx System (Neuro Probe, USA), as previously described^26,27^. Briefly, recombinant CXCL10 (Peprotech EC, UK) or siControl/siSOX17-treated culture supernatants, alone or in combination with the CXCR3 antagonist, AMG-487 (Selleckchem, USA) were dispensed into microplate wells of the ChemoTx System. L1.2-CXCR3 cells (pre-treated overnight with 10mM sodium butyrate) were seeded onto each filter of the ChemoTx membrane at density of 2 × 10^5^ cells/filter. The plate was then incubated in a humidified chamber for 5 h at 37 °C, 5% CO_2_. The number of cells migrating into the lower chamber was determined using the CellTiter-Glo^®^ Luminescent Cell Viability Assay (Promega, UK) with the use of a TopCount^®^ NXT Microplate Scintillation and Luminescence Counter, whereby luminescence was proportional to the number of cells (degree of migration). Data are expressed as the fold migration compared to migration buffer alone (Chemotactic index).

### Proteomics analysis

Plasma proteomic data (SomaScan v4; SomaLogic, USA) were available from a recent proteomics study by Rhodes et al., 2022 and Walters et al., 2023 ^3,28^. Therein, peripheral venous blood samples were acquired from healthy controls and idiopathic-PAH (IPAH) and heritable-PAH (HPAH) patients, recruited to the UK PAH Cohort study (East of England Research Ethics Committee [REC] 13/EE/0203). All subjects gave written, informed consent and all procedures were carried out in accordance with the relevant guidelines and regulations. Data are presented as a Z-score of healthy controls. Analyses were completed in R with RStudio version 1.4.1106.

## Results

### *SOX17* loss raises mRNA of CXCL10 and CXCL11 and other PAH-related pro-inflammatory factors

**Fig. 1:**
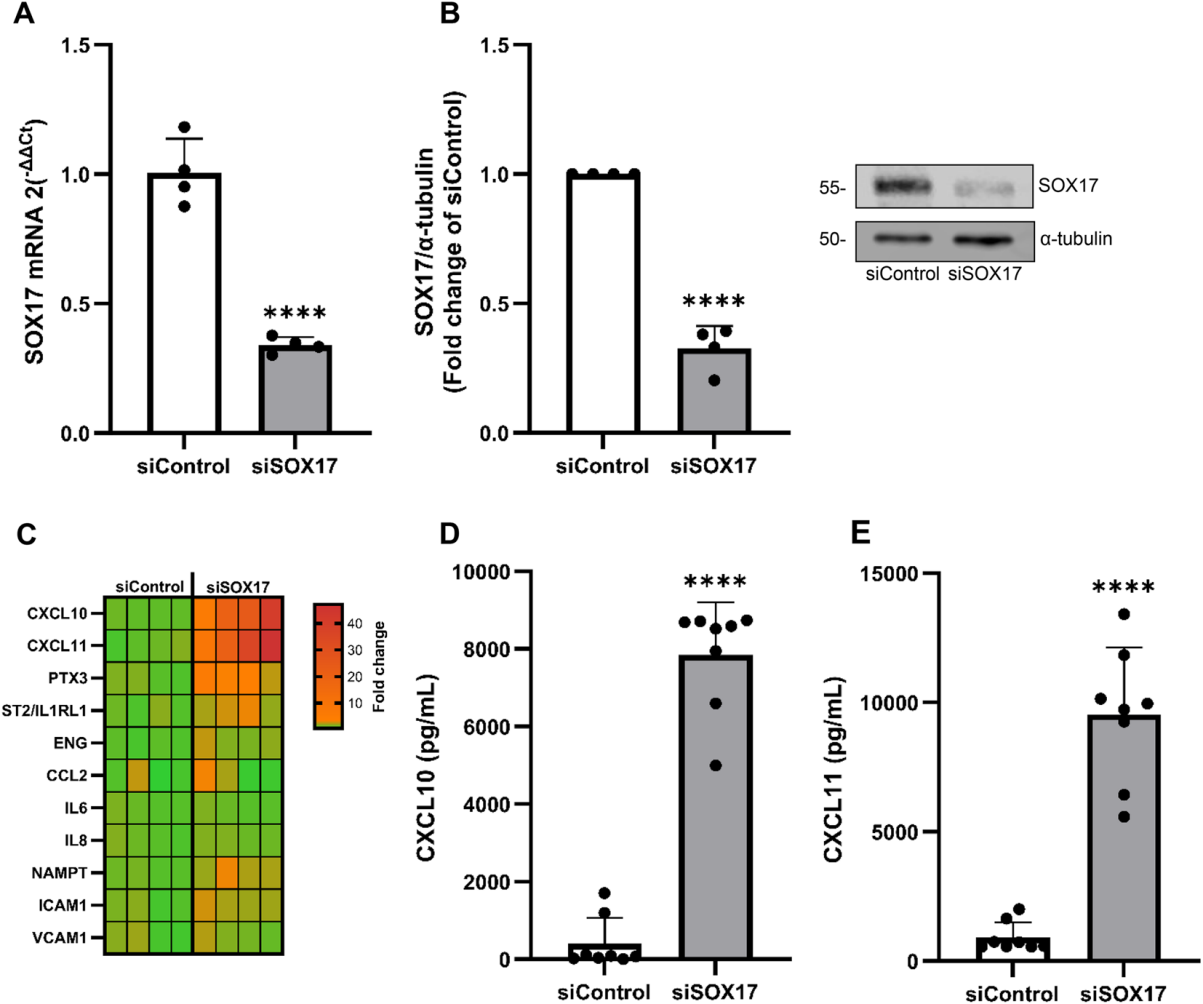
Endothelial SOX17 deficiency upregulates established PAH inflammatory mediators in particular CXCL10 and CXCL11. (**A**) confirmation of SOX17 mRNA knockdown post-siRNA transfection in HPAECs, normalised to GAPDH/ACTB and expressed relative to siControl. (**B**) SOX17 expression following gene knockdown in HPAECs (normalized to α-tubulin), with representative immunoblots (molecular weight in kDa is shown). (**C**) Heatmap displaying RT-qPCR data (2^−ΔΔCT^) of selected PAH-associated cytokines and chemokines in SOX17 knockdown HPAECs versus siControl. (**D**, **E**) soluble expression of CXCL10 and CXCL11 release in supernatants obtained from cultured siControl and siSOX17-treated HPAECs as determined by ELISA. ****P ≤ 0.0001 comparisons with siControl. Data are presented as mean + SEM and analysed by student’s t-test (*n* = 4–8).

In order to determine whether functional SOX17 loss induces cytokine and chemokine secretion, HPAECs were subjected to siRNA-induced *SOX17* knockdown (Fig. 1A-B), following which culture supernatants were exposed to a semi-quantitative antibody array. CXCL11 (17-fold), CXCL10 (3.3-fold), ST2/IL1RL1 (2.6-fold), pentraxin-3 (PTX3) (2.8-fold) and MCP1 (1.8-fold) were upregulated, whereas endoglin (ENG) was downregulated by approximately 50%, compared with siControl counterparts (Supplementary Fig. 1). Subsequently, these differentially expressed markers were validated using RT-qPCR of which *CXCL10* (23-fold), *CXCL11* (28-fold), *ST2/IL1RL1* (2-fold), *PTX3* (3-fold) and *ENG* (1.5-fold) were shown to be significantly (*P* ≤ 0.05) upregulated in siSOX17-treated cells compared with siControl (Fig. 1C). In addition, gene expression of other common PAH-associated cytokines, chemokine and adhesion molecules (*IL6*,* IL8*,* NAMPT*,* ICAM1 and VCAM1*) were assayed in *SOX17*-knockdown HPAECs, of which *NAMPT* (2-fold, *P* ≤ 0.01) and *ICAM1* (1.9-fold, *P* ≤ 0.05) were significantly overexpressed (Fig. 1C). *CXCR3*, the CXCL10 and CXCL11 receptor, was unchanged in SOX17 knockdown HPAECs (Supplementary Fig. 2).

### SOX17-deficient HPAECs markedly release soluble CXCL10 and CXCL11

Given the identification of marked CXCL10 and CXCL11 mRNA production, soluble protein release of these markers was confirmed via ELISA, of which both were shown to be significantly (*P* ≤ 0.0001) increased in siSOX17 supernatants compared with siControl (Fig. 1D and E).

### Plasma CXCL10 is elevated in PAH patients with pathogenic *SOX17* rare variants

**Fig. 2:**
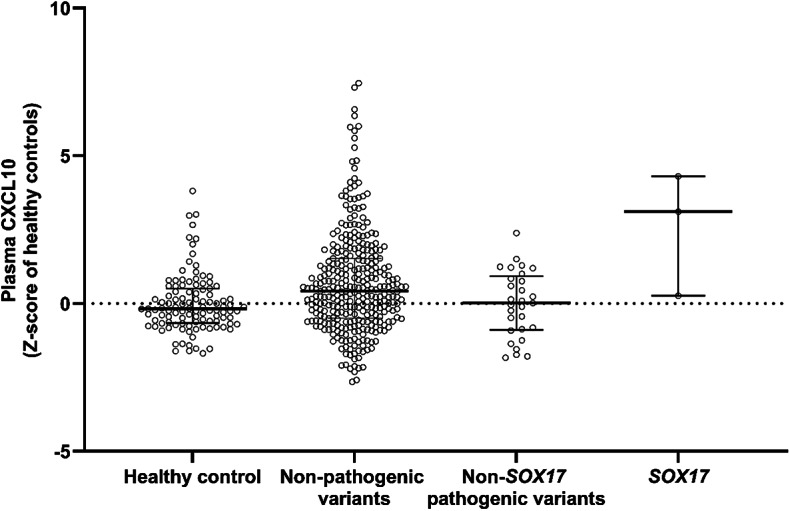
Plasma CXCL10 levels are raised in PAH particularly in carriers of pathogenic SOX17 rare variants. CXCL10 plasma levels in healthy controls (*n* = 108) and PAH patients: without known pathogenic variants (“Non-pathogenic variants”; *n* = 327), in carriers of pathogenic SOX17 rare variants (“*SOX17*”; *n* = 3) and in carriers with other rare pathogenic variants, namely *ACVRL1*,* ATP13A3*,* AQP1*,* EIF2AK4*,* GDF2*,* KCNK3*,* SMAD9* and *TBX4* (“Non-*SOX17* pathogenic variants”; *n* = 29). Data are presented as a Z-score of healthy controls.

Plasma proteomic data from PAH patients without known pathogenic variants (*n* = 327), shows that CXCL10 levels are mildly raised compared with healthy controls (*n* = 108; z-scores = 0.42 vs. -0.18; Fig. 2). Plasma CXCL10 in PAH patients bearing pathogenic *SOX17* rare variants (*n* = 3) is substantially raised (versus healthy controls), in two patients and slightly in the third (Z-scores = 4.3, 3.1, and 0.26), albeit a very limited sample size (Fig. 2). Interestingly, in PAH patients with non-*SOX17* pathogenic rare variants (*n* = 29; *ACVRL1*,* ATP13A3*,* AQP1*,* EIF2AK4*,* GDF2*,* KCNK3*,* SMAD9* and *TBX4*) CXCL10 plasma expression did not differ with healthy controls (Z-score = 0.01). Protein levels for CXCL11 were not available.

### NF-κB p65 activity is raised in SOX17-deficient HPAECs

**Fig. 3:**
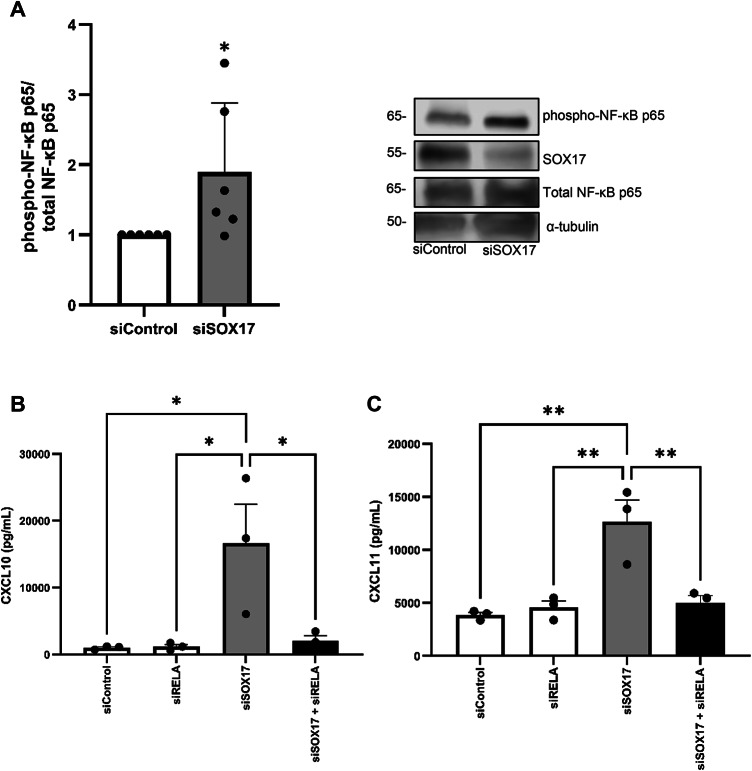
NF-κB p65 activity is increased in SOX17 knockdown HPAECs which drives excessive CXCL10 and CXCL11. (**A**) phospho-NF-κB p65 expression in siControl and siSOX17-treated HPAECs with representative immunoblots (molecular weight in kDa is shown). Original blots/gels are presented in Supplementary Fig. 4. (**B**) Measurements of CXCL10 and CXCL11 release in HPAECs transfected with siRELA, siSOX17, siSOX17 in combination with siRELA and non-targeting control (siControl), as determined by ELISA. *P ≤ 0.05, **P ≤ 0.01 comparisons with siControl or as indicated. Data are presented as mean + SEM and analysed by student’s t-test or one-way ANOVA with Tukey multiple-comparisons test. (*n* = 3–6).

NF-κB is an important transcriptional regulator of pro-inflammatory mediator production, and indeed its activity is increased in the pulmonary vasculature of PAH patients^8^. We therefore assayed levels of phospho-NF-κB p65 in *SOX17* knockdown HPAECs whereby we show that it is significantly (*P* ≤ 0.05) increased 1.9-fold in comparison with siControl-treated HPAECs (Fig. 3A).

### Abnormal CXCL10 and CXCL11 levels are instigated by aberrant NF-κB p65 activity in SOX17-deficient HPAECs

Subsequently, in order to establish whether the excess release of CXCL10 and CXCL11 was a consequence of the observed elevated NF-κB p65 activity, co-knockdown of *SOX17* and *RELA* (NF-κB p65 gene) were performed using siRNA (Supplementary Fig. 3). In these experiments, siSOX17/siRELA prevented siSOX17-induced CXCL10 and CXCL11 release in HPAECs (Fig. 3B and C).

### SOX17 deficiency in HPAECs drives chemotaxis of CXCR3 transfectants

**Fig. 4:**
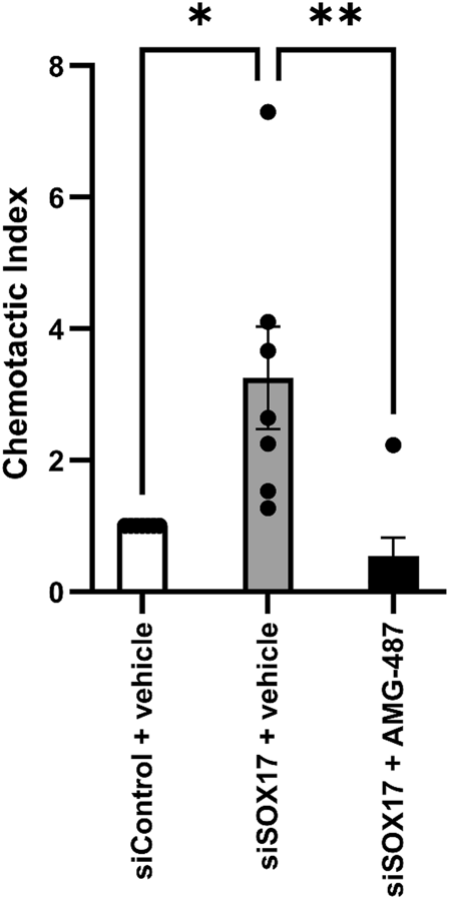
SOX17-knockdown drives chemotaxis of L1.2-CXCR3 transfectants via CXCR3. (**A**) The chemotactic response of L1.2-CXCR3 transfectants to siControl and siSOX17 (with and without AMG-487) matched culture supernatants. *P ≤ 0.05, **P ≤ 0.01 comparisons as indicated. Data are presented as mean + SEM and analysed by one-way ANOVA with Tukey multiple-comparisons test. (*n* = 7).

Given that CXCL10 and CXCL11 are recognised for their chemotactic activity at the chemokine receptor, CXCR3, we examined whether supernatants from *SOX17* knockdown HPAECs were capable of mediating chemotaxis of L1.2-CXCR3 transfectants using an in vitro modified Boyden chamber^18,21^. In a pilot experiment, varying concentrations of CXCL10 led to a bell-shaped dose-response in Chemotactic Index, normalized to buffer which peaked at 10nM (Supplementary Fig. 5A). Separately, migration of L1.2-CXCR3 transfectants to 1 nM CXCL10 alone resulted in a 32-fold increase in migration compared with buffer (Supplementary Fig. 5B). This was ameliorated in the presence of the CXCR3 antagonist AMG-487, thus confirming functional CXCR3 expression in the L1.2-CXCR3 cells (Supplementary Fig. 5B). Finally, *SOX17* knockdown supernatants engendered a significant increase (3.3-fold) in L1.2-CXCR3 cell migration compared with siControl supernatants (Fig. 4). This increase in migration was significantly attenuated in the presence of AMG-487.

## Discussion

This study shows for the first time that SOX17 deficiency in pulmonary artery endothelial cells, triggers excess production of CXCL10 and CXCL11 via NF-κB p65 activation. We validate these findings (in part) in patients, by providing evidence for substantially raised CXCL10 levels in a limited cohort of SOX17-PAH patients versus controls. We further show that supernatants from SOX17-deplete HPAECs instigate enhanced L1.2-CXCR3 transfectant migration via CXCR3, the receptor for CXCL10 and CXCL11. This may have implications for lymphocyte recruitment and would support a role for pro-inflammatory mechanisms of remodeling in PAH due to functional SOX17 loss.

CXCL10 and CXCL11 belong to the CXC family of chemokines, secreted predominantly by endothelial cells, monocytes and fibroblasts in response to interferon-gamma (IFN-γ)^17^. Among the many functional roles attributed to these cytokines, they are noted for orchestrating immune cell recruitment and differentiation, angiogenesis and tumour proliferation^17^. Indeed, CXCL10 has an established association in the pathophysiology of pulmonary hypertension (PH) where it is shown to be raised in the plasma of (IPAH) patients and also correlates with BNP and right ventricular dysfunction^11^. Further, in both SSc-PAH and chronic thromboembolic PH, serum expression of CXCL10 is raised and correlates with biomarkers of disease (BNP) and pulmonary hemodynamics^12,29^. In vitro, CXCL10 is pro-proliferative in human pulmonary artery smooth muscle cells and impairs HPAEC tube network formation^14^. Given our identification of marked CXCL10 and CXCL11 secretion from *SOX17*-silenced HPAECs together with elevated plasma CXCL10 in SOX17-PAH patients, it is plausible that these chemokines may feature as mediators of pulmonary vascular remodeling in SOX17-PAH. Further, given that SOX17 is primarily localised to the endothelium in the lung, it is therefore possible that the pulmonary endothelium may be a significant source of aberrant CXCL10 and CXCL11 secretion under conditions of impaired SOX17 in patients, although further studies in vivo are needed^2^.

We demonstrate that NF-κB p65 activity is raised following the silencing of *SOX17* in HPAECs. Indeed, aberrant NF-κB activation is a prominent pathophysiological driver of PAH. The pulmonary artery endothelium of IPAH patients, is characterized by significantly enhanced NF-κB expression and further, direct inhibition of NF-κB ameliorates MCT-induced PH in mice^8,30^. Whilst we have not explored a mechanism for the observed induction of NF-κB in our study, it is notable that Zou et al., 2023, describe that isolated exosomes from SOX17-overexpressing HPAECs prevent hypoxia- and TNFα-induced nuclear NF-κB p65 upregulation in HPAECs^7^. These effects, were thought to be engendered by the internalization of ‘vasculoprotective’ exosomal miRNAs, namely, miR-224-5p and miR-361-3p, otherwise shown to be reduced in expression following *SOX17* knockdown in the same cells^7^. Therefore, it is possible that SOX17 deficiency in the disease, may induce a dysfunctional pro-inflammatory endothelial cell phenotype, as already observed in PAH. This is further supported given that knockdown of *SOX17* in our model, increases NF-κB p65 activity in HPAECs, triggering the overproduction of CXCL10 and CXCL11. Furthermore, we demonstrate upregulation (mRNA) of several inflammatory markers, *ST2/IL1RL1*,* PTX3*,* ENG*,* NAMPT* and *ICAM1*, all of which have previously been implicated in the pathophysiology of PAH^31–35^. Interestingly, IL33, the ligand for ST2 has previously been shown to upregulate SOX17 expression in pulmonary microvascular endothelial cells, suggesting that the increased *ST2/IL1RL1* shown in our model may be a potential compensatory mechanism for SOX17 impairment^36^. Whilst it is clear from our study that SOX17 status may influence NF-κB activation, it is unclear whether NF-κB may regulate the expression of SOX17. However, the Wnt/β-catenin signalling pathway is an established regulator of SOX17^37^. Additionally, NF-κB has been shown to both enhance and suppress Wnt/β-catenin activity, which could potentially influence SOX17 expression as a downstream effect^38^.

Given the otherwise established role of CXCL10 and CXCL11 in immune cell chemotaxis, we subsequently showed that *SOX17* knockdown supernatants facilitated increased L1.2-CXCR3 transfectant migration compared with siControl-treated counterparts^18,22^. Indeed, this effect was abolished in the presence of AMG-487, suggesting that the chemokines, CXCL10 and CXCL11, enriched in the siSOX17 supernatants, mediated the observed effects. Our findings compliment other studies; whereby adult mice with inducible endothelial-specific *SOX17* deletion present with significantly elevated perivascular accumulation of CD45^+^ and CD11b^+^ cells^5,6^. Notably the former is a pan-leukocyte marker and the latter is thought to be expressed on myeloid-lineage cells, some of which include monocytes/macrophages, neutrophils, eosinophils, and a subset of T- and B cells^39,40^. Taken together, and considering that T cell and B cell pulmonary vascular infiltration is associated with the pathobiology of PAH, our data may suggest a possible role for aberrant lymphocyte chemotaxis driven by a CXCL/CXCR3 axis in SOX17-PAH^9^.

Further studies are needed to examine plasma CXCL11 levels in patients with SOX17-PAH to address whether their expression is changed with *SOX17* loss. Indeed, whilst we ascertained CXCL10 levels in the plasma of these patients, larger patient cohorts with pathogenic *SOX17* rare variants are required to obtain conclusive data. Certainly, future investigations should histologically assess leukocyte adhesion/infiltration as well as NF-κB levels in SOX17-PAH lung tissue. In addition, the potential role of NF-κB as a regulator of SOX17 should be explored. Indeed, these notions also serve as apparent limitations of our study. Further, normalization of assayed soluble CXCL10/CXCL11 concentrations to total protein content was not carried out.

In summary, we provide the first experimental association of pulmonary endothelial SOX17 deficiency and NF-κB p65-dependent CXCL10 and CXCL11 upregulation, together with increased CXCR3-driven cell migration in vitro. These findings may indicate a pro-inflammatory mechanism of endothelial dysfunction in SOX17-PAH, which may have implications for the initiation and/or progression of PAH in patients with these pathogenic rare variants.

## Supplementary Information

Below is the link to the electronic supplementary material.

Supplementary Material 1

## Data Availability

The datasets used and/or analysed in the current study are available from the corresponding author on reasonable request.

## Abbreviations

SOX17: SRY-box transcription factor 17
CXCL: Chemokine (C-X-C motif) ligand
PAH: Pulmonary arterial hypertension
NF-κB: Nuclear factor kappa-light-chain-enhancer of activated B cells

## References

[1] Hassoun, P. M. Pulmonary arterial hypertension. *N Engl. J. Med.* **385**, 2361–2376. 10.1056/NEJMra2000348 (2021). 34910865 10.1056/NEJMra2000348

[2] Graf, S. et al. Identification of rare sequence variation underlying heritable pulmonary arterial hypertension. *Nat. Commun.* **9**, 1416. 10.1038/s41467-018-03672-4 (2018). 29650961 10.1038/s41467-018-03672-4 PMC5897357

[3] Walters, R. et al. SOX17 enhancer variants disrupt transcription factor binding and enhancer inactivity drives pulmonary hypertension. *Circulation* **147**, 1606–1621. 10.1161/CIRCULATIONAHA.122.061940 (2023). 37066790 10.1161/CIRCULATIONAHA.122.061940 PMC7614572

[4] Rhodes, C. J. et al. Genetic determinants of risk in pulmonary arterial hypertension: international genome-wide association studies and meta-analysis. *Lancet Respir Med.* **7**, 227–238. 10.1016/S2213-2600(18)30409-0 (2019). 30527956 10.1016/S2213-2600(18)30409-0 PMC6391516

[5] Park, C. S. et al. Sox17 deficiency promotes pulmonary arterial hypertension via HGF/c-Met signaling. *Circ. Res.* **131**, 792–806. 10.1161/CIRCRESAHA.122.320845 (2022). 36205124 10.1161/CIRCRESAHA.122.320845 PMC9612711

[6] Yi, D. et al. E2F1 mediates SOX17 Deficiency-Induced pulmonary hypertension. *Hypertension* **80**, 2357–2371. 10.1161/HYPERTENSIONAHA.123.21241 (2023). 37737027 10.1161/HYPERTENSIONAHA.123.21241 PMC10591929

[7] Zou, X. et al. SOX17 is a critical factor in maintaining endothelial function in pulmonary hypertension by an Exosome-Mediated autocrine manner. *Adv. Sci. (Weinh)*. **10**, e2206139. 10.1002/advs.202206139 (2023). 36919784 10.1002/advs.202206139 PMC10190640

[8] Price, L. C. et al. Nuclear factor kappa-B is activated in the pulmonary vessels of patients with end-stage idiopathic pulmonary arterial hypertension. *PLoS One*. **8**, e75415. 10.1371/journal.pone.0075415 (2013). 24124488 10.1371/journal.pone.0075415 PMC3790752

[9] Hu, Y., Chi, L., Kuebler, W. M. & Goldenberg, N. M. Perivascular inflammation in pulmonary arterial hypertension. *Cells* **9**10.3390/cells9112338 (2020). 10.3390/cells9112338 PMC7690279 33105588

[10] Itoh, T. et al. Increased plasma monocyte chemoattractant protein-1 level in idiopathic pulmonary arterial hypertension. *Respirology* **11**, 158–163. 10.1111/j.1440-1843.2006.00821.x (2006). 16548900 10.1111/j.1440-1843.2006.00821.x

[11] Yang, T. et al. Increased levels of plasma CXC-Chemokine ligand 10, 12 and 16 are associated with right ventricular function in patients with idiopathic pulmonary arterial hypertension. *Heart Lung*. **43**, 322–327. 10.1016/j.hrtlng.2014.04.016 (2014). 24856224 10.1016/j.hrtlng.2014.04.016

[12] George, P. M. et al. Evidence for the involvement of type I interferon in pulmonary arterial hypertension. *Circ. Res.* **114**, 677–688. 10.1161/CIRCRESAHA.114.302221 (2014). 24334027 10.1161/CIRCRESAHA.114.302221 PMC4006084

[13] Burdick, M. D. et al. CXCL11 attenuates bleomycin-induced pulmonary fibrosis via Inhibition of vascular remodeling. *Am. J. Respir Crit. Care Med.* **171**, 261–268. 10.1164/rccm.200409-1164OC (2005). 15502109 10.1164/rccm.200409-1164OC

[14] Zabini, D. et al. Angiostatic factors in the pulmonary endarterectomy material from chronic thromboembolic pulmonary hypertension patients cause endothelial dysfunction. *PLoS One*. **7**, e43793. 10.1371/journal.pone.0043793 (2012). 22916307 10.1371/journal.pone.0043793 PMC3423379

[15] Zhang, Y. et al. CXCL11 promotes self-renewal and tumorigenicity of alpha2delta1(+) liver tumor-initiating cells through CXCR3/ERK1/2 signaling. *Cancer Lett.* **449**, 163–171. 10.1016/j.canlet.2019.02.016 (2019). 30771435 10.1016/j.canlet.2019.02.016

[16] Brandt, E. F. et al. Chemokine CXCL10 modulates the tumor microenvironment of Fibrosis-Associated hepatocellular carcinoma. *Int. J. Mol. Sci.* **23**10.3390/ijms23158112 (2022). 10.3390/ijms23158112 PMC9329882 35897689

[17] Tokunaga, R. et al. CXCL10, CXCL11/CXCR3 axis for immune activation - A target for novel cancer therapy. *Cancer Treat. Rev. 63*. **CXCL9**, 40–47. 10.1016/j.ctrv.2017.11.007 (2018). 10.1016/j.ctrv.2017.11.007 PMC5801162 29207310

[18] Taub, D. D. et al. Recombinant human interferon-inducible protein 10 is a chemoattractant for human monocytes and T lymphocytes and promotes T cell adhesion to endothelial cells. *J. Exp. Med.* **177**, 1809–1814. 10.1084/jem.177.6.1809 (1993). 8496693 10.1084/jem.177.6.1809 PMC2191047

[19] Tomita, K. et al. CXCL10-Mediates macrophage, but not other innate immune Cells-Associated inflammation in murine nonalcoholic steatohepatitis. *Sci. Rep.* **6**, 28786. 10.1038/srep28786 (2016). 27349927 10.1038/srep28786 PMC4923862

[20] Petrovic-Djergovic, D. et al. CXCL10 induces the recruitment of monocyte-derived macrophages into kidney, which aggravate puromycin aminonucleoside nephrosis. *Clin. Exp. Immunol.* **180**, 305–315. 10.1111/cei.12579 (2015). 25561167 10.1111/cei.12579 PMC4408165

[21] Cole, K. E. et al. Interferon-inducible T cell alpha chemoattractant (I-TAC): a novel non-ELR CXC chemokine with potent activity on activated T cells through selective high affinity binding to CXCR3. *J. Exp. Med.* **187**, 2009–2021. 10.1084/jem.187.12.2009 (1998). 9625760 10.1084/jem.187.12.2009 PMC2212354

[22] Torraca, V. et al. The CXCR3-CXCL11 signaling axis mediates macrophage recruitment and dissemination of mycobacterial infection. *Dis. Model. Mech.* **8**, 253–269. 10.1242/dmm.017756 (2015). 25573892 10.1242/dmm.017756 PMC4348563

[23] Meiser, A. et al. The chemokine receptor CXCR3 is degraded following internalization and is replenished at the cell surface by de Novo synthesis of receptor. *J. Immunol.* **180**, 6713–6724. 10.4049/jimmunol.180.10.6713 (2008). 18453591 10.4049/jimmunol.180.10.6713 PMC2556381

[24] Xanthou, G., Williams, T. J. & Pease, J. E. Molecular characterization of the chemokine receptor CXCR3: evidence for the involvement of distinct extracellular domains in a multi-step model of ligand binding and receptor activation. *Eur. J. Immunol.* **33**, 2927–2936. 10.1002/eji.200324235 (2003). 14515277 10.1002/eji.200324235

[25] Shao, D. et al. Nuclear IL-33 regulates soluble ST2 receptor and IL-6 expression in primary human arterial endothelial cells and is decreased in idiopathic pulmonary arterial hypertension. *Biochem. Biophys. Res. Commun.* **451**, 8–14. 10.1016/j.bbrc.2014.06.111 (2014). 25003325 10.1016/j.bbrc.2014.06.111

[26] Vaidehi, N., Pease, J. E. & Horuk, R. Modeling small molecule-compound binding to G-protein-coupled receptors. *Methods Enzymol.* **460**, 263–288. 10.1016/S0076-6879(09)05213-6 (2009). 19446730 10.1016/S0076-6879(09)05213-6

[27] Nedjai, B. et al. Small molecule chemokine mimetics suggest a molecular basis for the observation that CXCL10 and CXCL11 are allosteric ligands of CXCR3. *Br. J. Pharmacol.* **166**, 912–923. 10.1111/j.1476-5381.2011.01660.x (2012). 21895630 10.1111/j.1476-5381.2011.01660.x PMC3417418

[28] Rhodes, C. J. et al. Using the plasma proteome for risk stratifying patients with pulmonary arterial hypertension. *Am. J. Respir Crit. Care Med.* **205**, 1102–1111. 10.1164/rccm.202105-1118OC (2022). 35081018 10.1164/rccm.202105-1118OC PMC9851485

[29] Hong, C. et al. CXCL10 levels in diagnosis and improved hemodynamics in patients with chronic thromboembolic pulmonary hypertension undergoing balloon pulmonary angioplasty. *Pulm Circ.* **12**, e12091. 10.1002/pul2.12091 (2022). 35685949 10.1002/pul2.12091 PMC9171940

[30] Li, L., Wei, C., Kim, I. K., Janssen-Heininger, Y. & Gupta, S. Inhibition of nuclear factor-kappaB in the lungs prevents monocrotaline-induced pulmonary hypertension in mice. *Hypertension* **63**, 1260–1269. 10.1161/HYPERTENSIONAHA.114.03220 (2014). 24614212 10.1161/HYPERTENSIONAHA.114.03220

[31] Tielemans, B. et al. Cytokines trigger disruption of endothelium barrier function and p38 MAP kinase activation in BMPR2-silenced human lung microvascular endothelial cells. *Pulm Circ.* **9**, 2045894019883607. 10.1177/2045894019883607 (2019). 31692724 10.1177/2045894019883607 PMC6811766

[32] O’Neill, C. L. et al. Endothelial cell-derived pentraxin 3 limits the vasoreparative therapeutic potential of Circulating angiogenic cells. *Cardiovasc. Res.* **112**, 677–688. 10.1093/cvr/cvw209 (2016). 27659714 10.1093/cvr/cvw209 PMC5157134

[33] Zheng, Y. G. et al. Plasma soluble ST2 levels correlate with disease severity and predict clinical worsening in patients with pulmonary arterial hypertension. *Clin. Cardiol.* **37**, 365–370. 10.1002/clc.22262 (2014). 25068163 10.1002/clc.22262 PMC6649535

[34] Malhotra, R. et al. Circulating angiogenic modulatory factors predict survival and functional class in pulmonary arterial hypertension. *Pulm Circ.* **3**, 369–380. 10.4103/2045-8932.110445 (2013). 24015338 10.4103/2045-8932.110445 PMC3757832

[35] Sun, X. et al. Direct extracellular NAMPT involvement in pulmonary hypertension and vascular remodeling. Transcriptional regulation by SOX and HIF-2alpha. *Am. J. Respir Cell. Mol. Biol.* **63**, 92–103. 10.1165/rcmb.2019-0164OC (2020). 32142369 10.1165/rcmb.2019-0164OC PMC7328254

[36] Ha, E. H. et al. Endothelial Sox17 promotes allergic airway inflammation. *J Allergy Clin Immunol* **144**, 561–573 e566 (2019). 10.1016/j.jaci.2019.02.034 10.1016/j.jaci.2019.02.034 30928652

[37] Engert, S. et al. Wnt/beta-catenin signalling regulates Sox17 expression and is essential for organizer and endoderm formation in the mouse. *Development* **140**, 3128–3138. 10.1242/dev.088765 (2013). 23824574 10.1242/dev.088765

[38] Ma, B. & Hottiger, M. O. Crosstalk between Wnt/beta-Catenin and NF-kappaB signaling pathway during inflammation. *Front. Immunol.* **7**, 378. 10.3389/fimmu.2016.00378 (2016). 27713747 10.3389/fimmu.2016.00378 PMC5031610

[39] Christensen, J. E., Andreasen, S. O., Christensen, J. P. & Thomsen, A. R. CD11b expression as a marker to distinguish between recently activated effector CD8(+) T cells and memory cells. *Int. Immunol.* **13**, 593–600. 10.1093/intimm/13.4.593 (2001). 11282998 10.1093/intimm/13.4.593

[40] Griffin, D. O. & Rothstein, T. L. A small CD11b(+) human B1 cell subpopulation stimulates T cells and is expanded in lupus. *J. Exp. Med.* **208**, 2591–2598. 10.1084/jem.20110978 (2011). 22110167 10.1084/jem.20110978 PMC3244038

