# Supplementary material for: SOX17-silenced HPAECs upregulate NF-κB-induced CXCL10 and CXCL11: implications for lymphocyte chemotaxis in SOX17-PAH
Source: Sci Rep. 2025 Oct 1;15:34262. doi: 10.1038/s41598-025-16418-2 (PMC12488963; doi:10.1038/s41598-025-16418-2)
Supplement: Supplementary file 1 — Supplementary Material 1 [file 41598_2025_16418_MOESM1_ESM.docx]

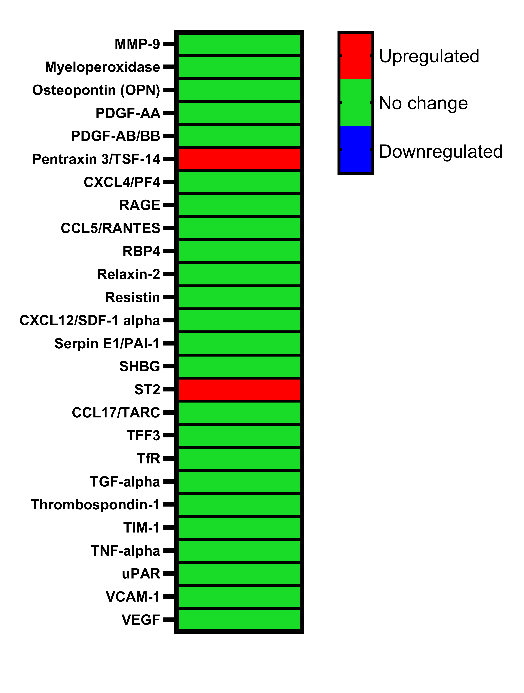

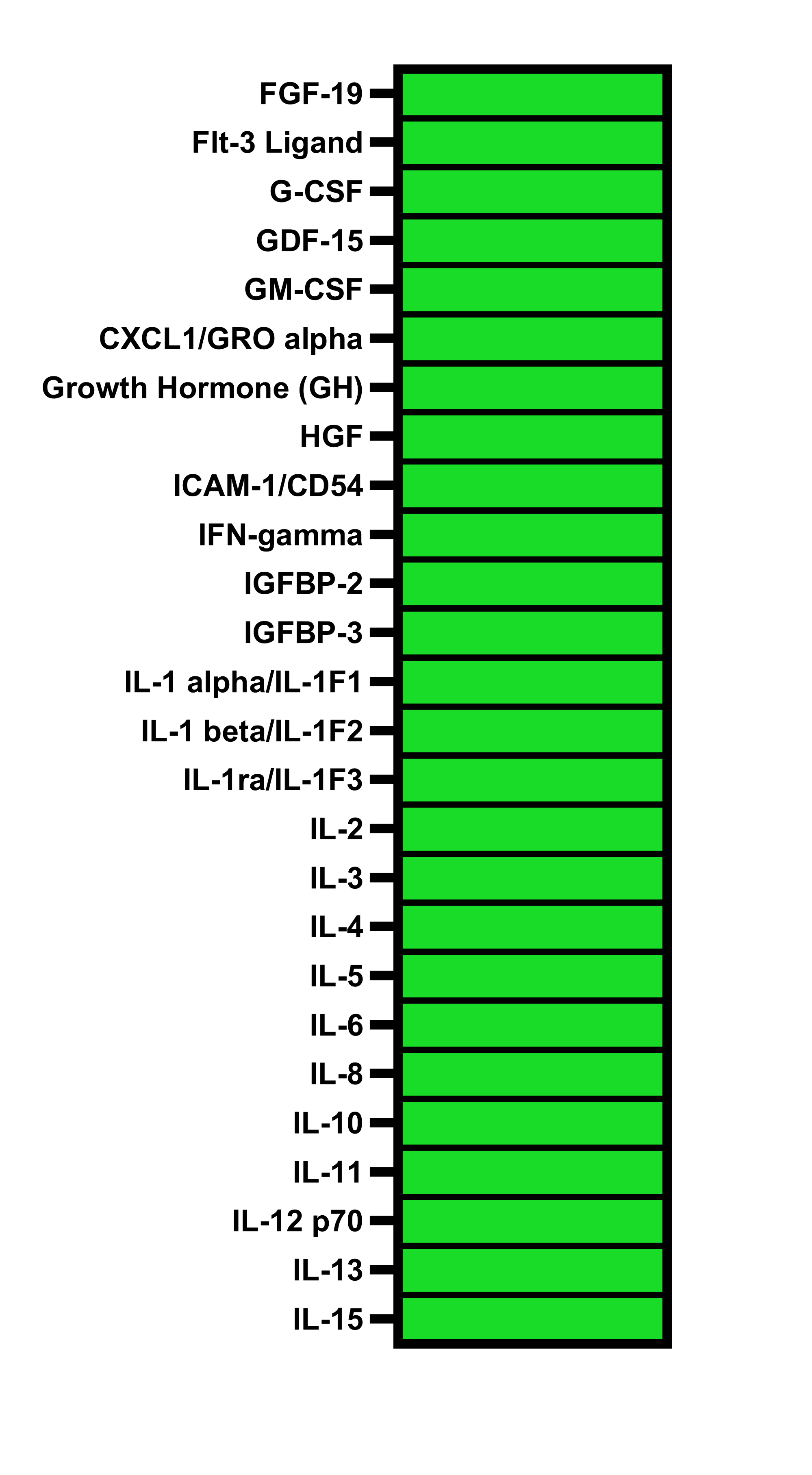

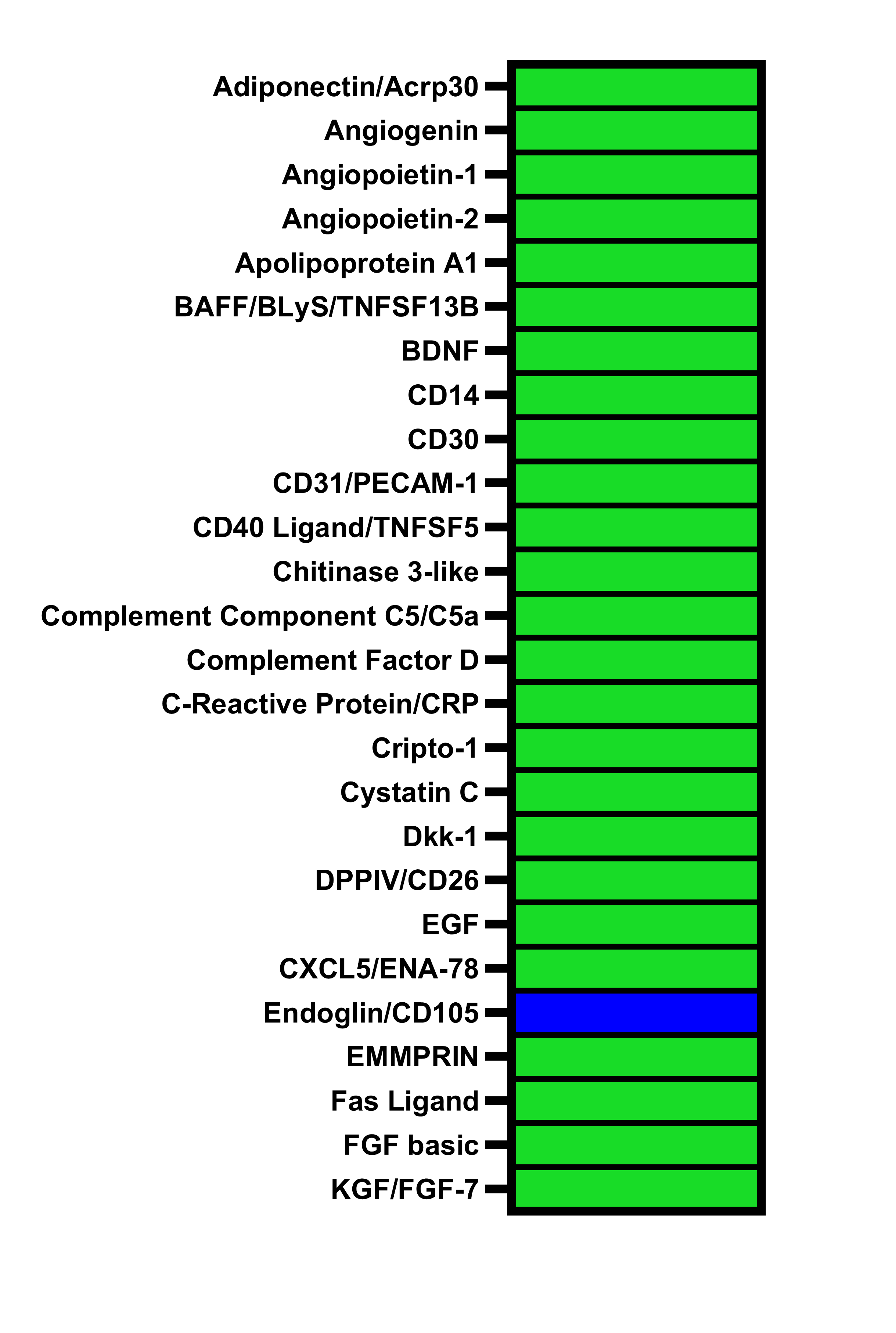

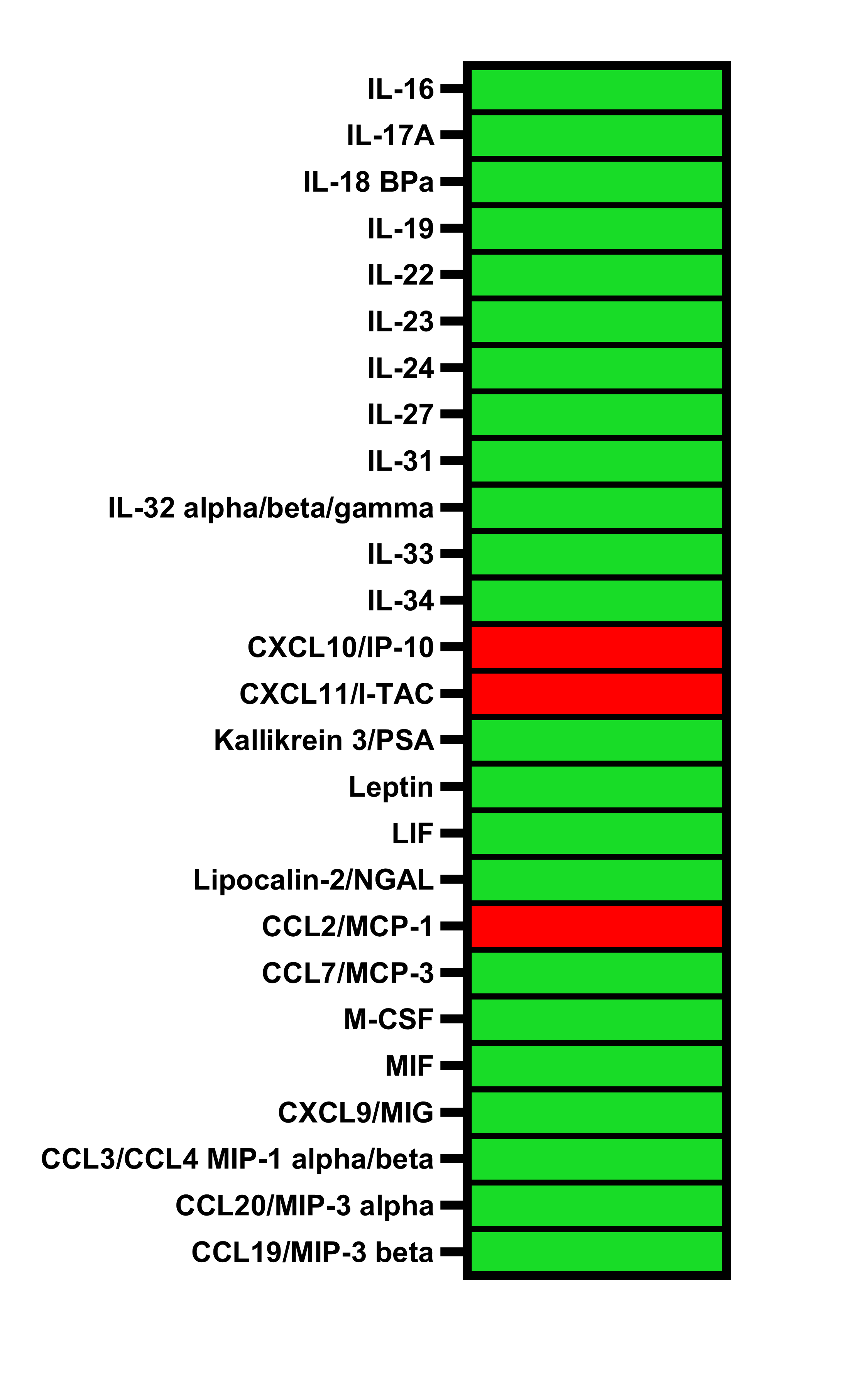


**Supplementary Figure 1. SOX17 deficiency in HPAECs drives release of inflammatory cytokines and chemokines.** Heatmap depicts differentially expressed soluble proteins released from siSOX17 HPAECs identified using a cytokine and chemokine array (n=1). Proteins are shown as upregulated (red), downregulated (blue) or unchanged (green) in siSOX17 supernatants versus siControl. Upregulated markers in siSOX17 supernatants included CXCL10 (3.3-fold), CXCL11 (17-fold), CCL2 (1.8-fold), pentraxin-3 (2.8-fold) and ST2 (2.6-fold) compared with siControl counterparts. Endoglin was shown to be downregulated 0.5-fold in SOX17-knockdown HPAEC supernatants versus siControl.

**
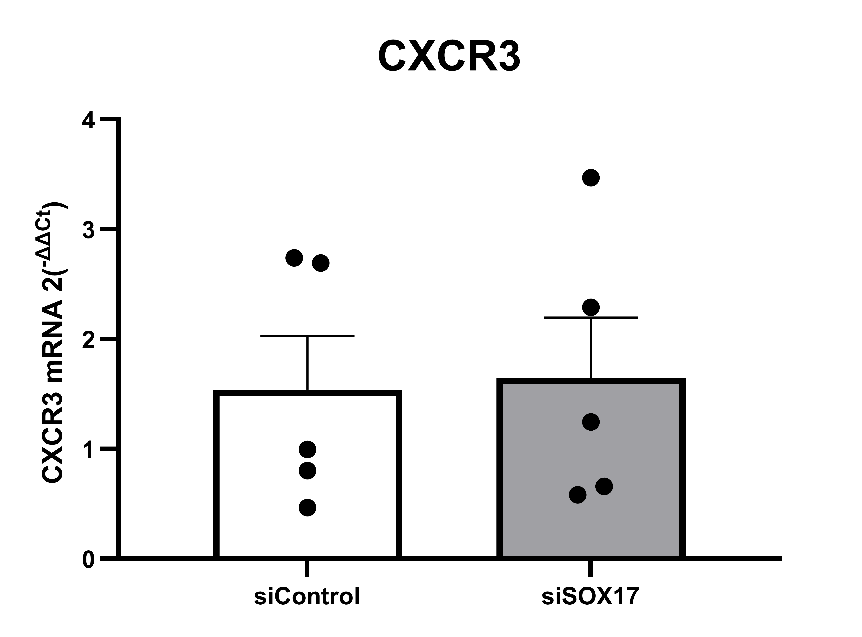
**

**Supplementary Figure 2. CXCR3 gene expression is unchanged post-SOX17 knockdown.** Quantification of CXCR3 mRNA post-SOX17 siRNA transfection (siSOX17) in HPAECs, normalised to GAPDH/ACTB and expressed relative to siControl (2^-ΔΔCT^). Data are presented as mean + SEM and analysed by student’s t-test (n=5).


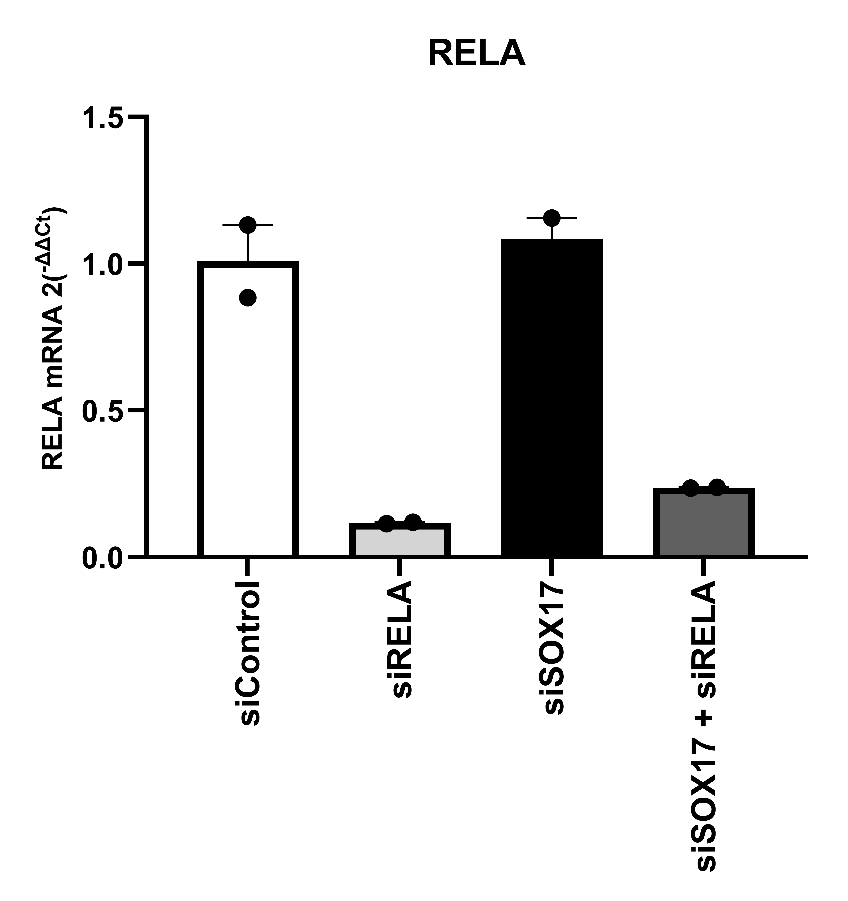


**Supplementary Figure 3. RELA gene expression following co-knockdown of RELA and SOX17.** Quantification of RELA mRNA post-siRNA transfection (as shown) in HPAECs, normalised to GAPDH/ACTB and expressed relative to siControl (2^-ΔΔCT^). Data are presented as mean + SEM (n=2).


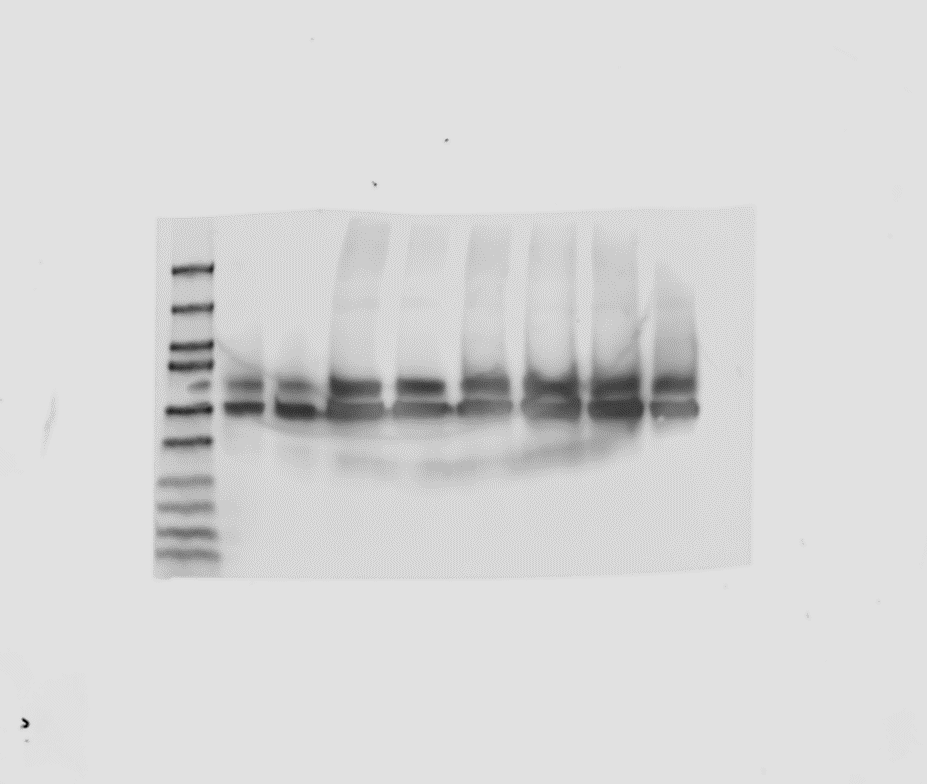


250 kDa

150 kDa

100 kDa

75 kDa

50 kDa

37 kDa

25 kDa

20 kDa

15 kDa

10 kDa

Total NF-κB p65

α-tubulin


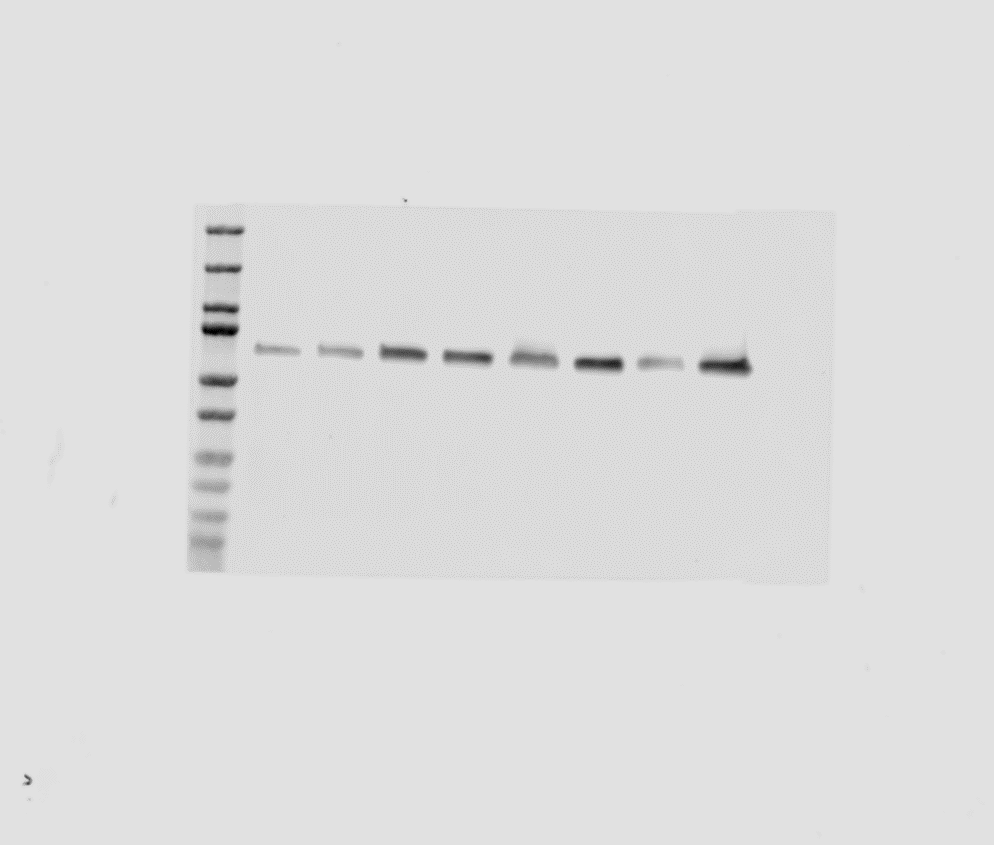


250 kDa

150 kDa

100 kDa

75 kDa

50 kDa

37 kDa

25 kDa

20 kDa

15 kDa

10 kDa

Phospho-NF-κB p65


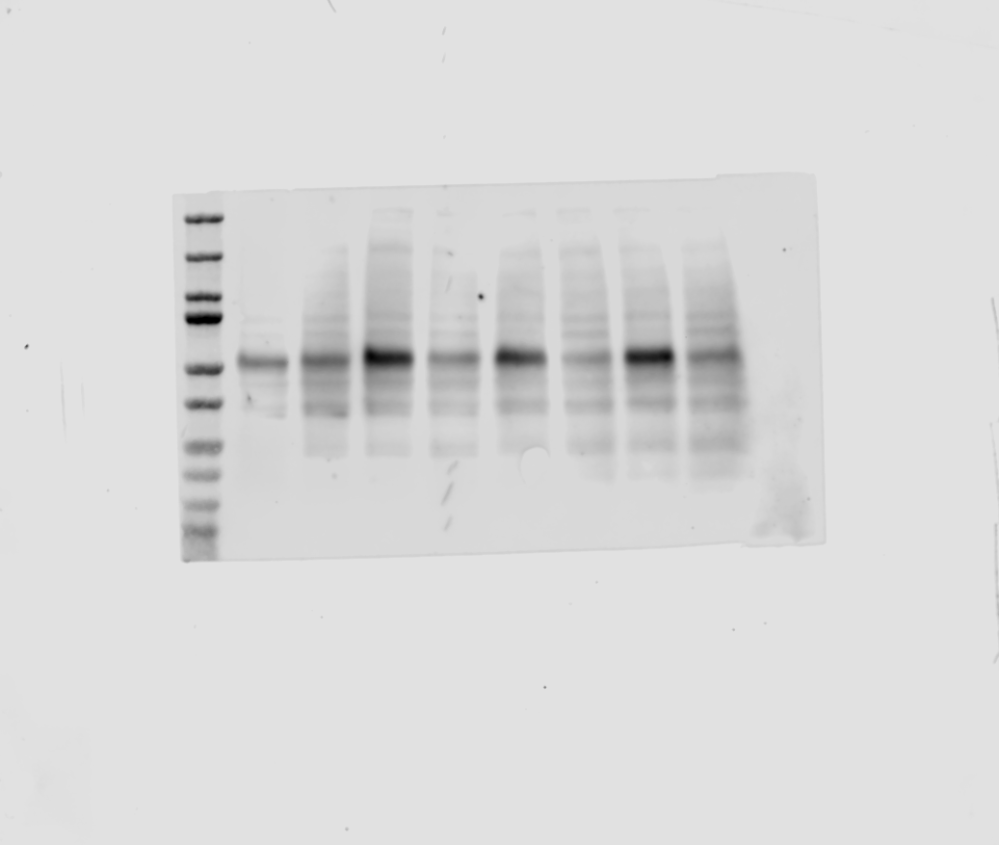


250 kDa

150 kDa

100 kDa

75 kDa

50 kDa

37 kDa

25 kDa

20 kDa

15 kDa

10 kDa

SOX17

S

S

S

S

C

C

C

C

C

S

C

S

C

S

C

S

S

C

C

C

S

S

S

C

**Supplementary Figure 4. Uncropped full-length blots of phospho-NF-κB p65, total NF-κB p65, α-tubulin and SOX17 (Figure 3).** Blots show expression of phospho-NF-κB p65, total NF-κB p65, α-tubulin and SOX17 (together with molecular weight markers) in siControl and siSOX17-treated HPAECs (denoted above as C and S, respectively) as shown and quantified in Figure 3.


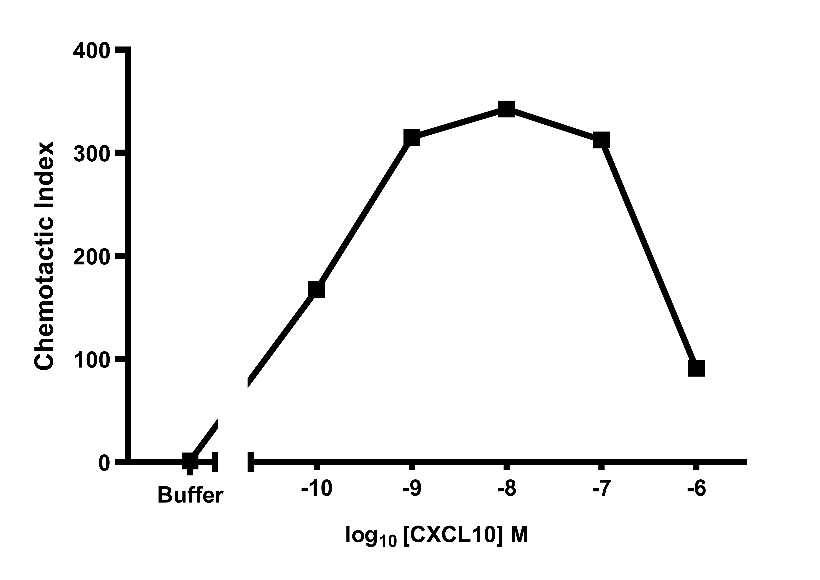

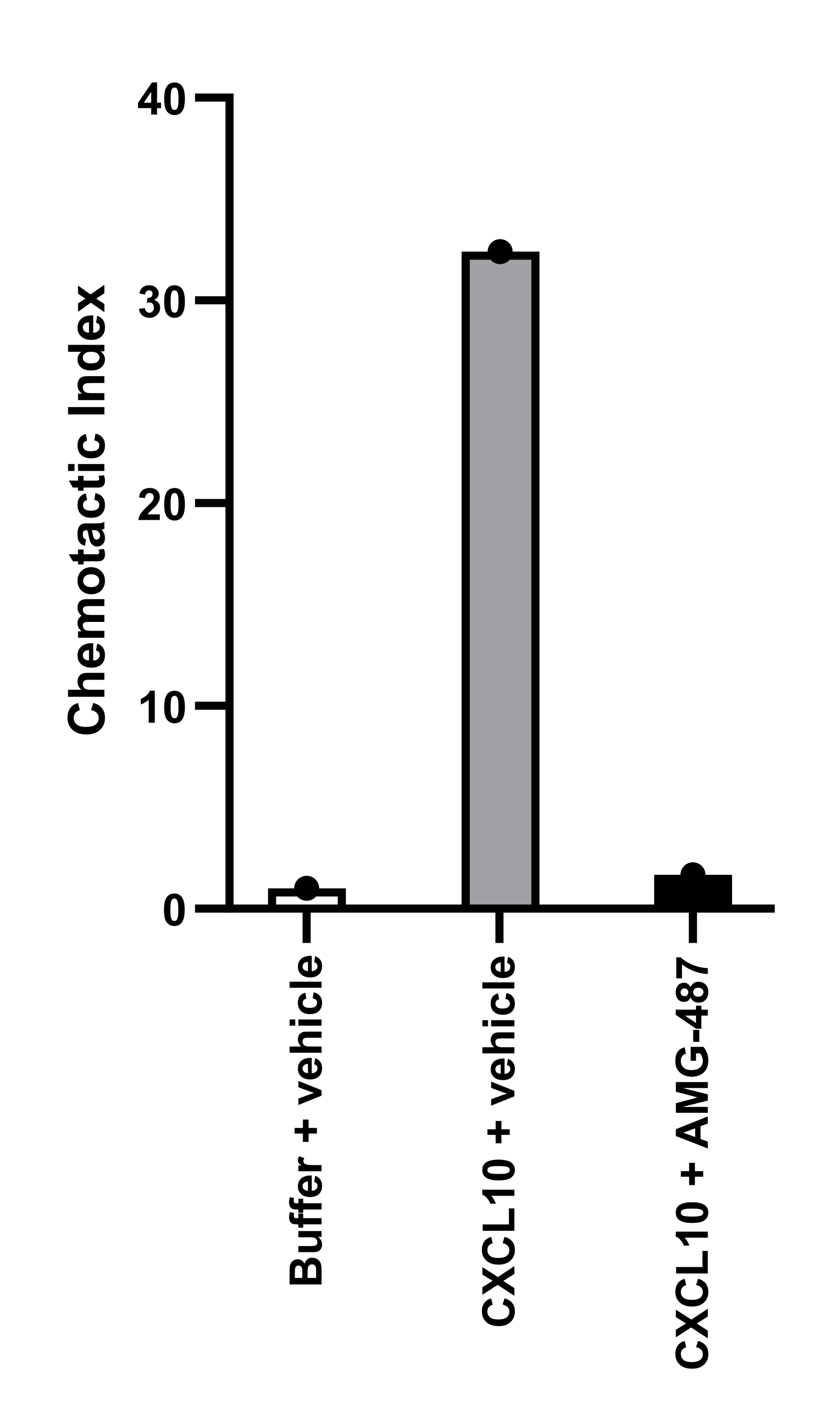


**A**

**B**

**Supplementary Figure 5. CXCL10-induced migration of L1.2-CXCR3 transfectants and inhibition with CXCR3 antagonist. (A)** The chemotactic response of L1.2-CXCR3 transfectants to increasing concentrations of CXCL10 (as shown), **(B)** CXCL10 (1nM) alone or combined with CXCR3 antagonist (AMG-487). (n=1).

| Target | Primer sequence (5’-3’) | |
| --- | --- | --- |
|  | Forward | Reverse |
| ACTA2 | AGATCAAGATCATTGCCCC | TTCATCGTATTCCTGTTTGC |
| CCL2 | GATCTCAGTGCAGAGGCTCG | TGCTTGTCCAGGTGGTCCAT |
| CXCL10 | AAAGCAGTTAGCAAGGAAAG | TCATTGGTCACCTTTTAGTG |
| CXCL11 | CTACAGTTGTTCAAGGCTTC | CACTTTCACTGCTTTTACCC |
| ENG | CTTCCTGGAGTTCCCAACG | GGTGCCATTTTGCTTGGA |
| GAPDH | ACAGTTGCCATGTAGACC | TTGAGCACAGGGTACTTTA |
| ICAM1 | GGCTGGAGCTGTTTGAGAAC | ACTGTGGGGTTCAACCTCTG |
| IL1RL1 | CACGGTCAAGGATGAGCAAG | GCAGAGCAAGTTAGGTTTGCG |
| IL6 | GCAGAAAAAGGCAAAGAATC | CTACATTTGCCGAAGAGC |
| IL8 | ACTGAGAGTGATTGAGAGTGGAC | AACCCTCTGCACCCAGTTTTC |
| NAMPT | AAGGGTTACAAGTTGCTGCC | AACAAAATTCCCTGCTGGCG |
| PTX3 | GGGACAAGCTCTTCATCATGCT | GTCGTCCGTGGCTTGCA |
| RELA | GCACAGATACCACCAAGACC | TCAGCCTCATAGAAGCCATC |
| SOX17 | GGACCGCACGGAATTTGAAC | GGACACCACCGAGGAAATGG |
| VCAM1 | TCAGATTGGAGACTCAGTCATGT | ACTCCTCACCTTCCCGCTC |

**Supplementary Table 1.** Forward and reverse RT-qPCR primer sequences used in this study.
